# A Flavonoid, Luteolin, Cripples HIV-1 by Abrogation of Tat Function

**DOI:** 10.1371/journal.pone.0027915

**Published:** 2011-11-30

**Authors:** Rajeev Mehla, Shalmali Bivalkar-Mehla, Ashok Chauhan

**Affiliations:** 1 Department of Pathology, Microbiology and Immunology, University of South Carolina School of Medicine, Columbia, South Carolina, United States of America; 2 Department of Pharmacology, Physiology and Neuroscience, University of South Carolina School of Medicine, Columbia, South Carolina, United States of America; Johns Hopkins School of Medicine, United States of America

## Abstract

Despite the effectiveness of combination antiretroviral treatment (cART) against HIV-1, evidence indicates that residual infection persists in different cell types. Intensification of cART does not decrease the residual viral load or immune activation. cART restricts the synthesis of infectious virus but does not curtail HIV-1 transcription and translation from either the integrated or unintegrated viral genomes in infected cells. All treated patients with full viral suppression actually have low-level viremia. More than 60% of treated individuals also develop minor HIV-1 –associated neurocognitive deficits (HAND) due to residual virus and immune activation. Thus, new therapeutic agents are needed to curtail HIV-1 transcription and residual virus. In this study, luteolin, a dietary supplement, profoundly reduced HIV-1 infection in reporter cells and primary lymphocytes. HIV-1inhibition by luteolin was independent of viral entry, as shown by the fact that wild-type and VSV–pseudotyped HIV-1 infections were similarly inhibited. Luteolin was unable to inhibit viral reverse transcription. Luteolin had antiviral activity in a latent HIV-1 reactivation model and effectively ablated both clade-B- and -C -Tat-driven LTR transactivation in reporter assays but had no effect on Tat expression and its sub-cellular localization. We conclude that luteolin confers anti–HIV-1 activity at the Tat functional level. Given its biosafety profile and ability to cross the blood-brain barrier, luteolin may serve as a base flavonoid to develop potent anti–HIV-1 derivatives to complement cART.

## Introduction

HIV-1 infection of the host cells proceeds with reverse transcription, viral DNA integration into the host genome, transcription, translation, proteolytic processing of viral proteins and subsequent assembly into nascent viral particles [1]. To a large extent, the introduction of combination antiretroviral treatment (cART) has curtailed viral replication below the detection limit (<50 copies/mL) and significantly reduced the devastating impact of HIV-1 [2]–[5]. cART works by blocking infection of susceptible new cells, while the decay rate of plasma virus is determined by the life span of previously infected cells [6]. However, given the presence of intact HIV-1 reservoirs, including quiescent CD4+ T lymphocytes, bone marrow and brain [7]–[8], as well as the development of viral escape mutants and drug resistance, viral replication goes unchecked by intensive therapy [9]–[14]. All treated patients with full viral suppression actually have low-level, steady-state viremia [10], [15]–[16].

Given the long duration of treatment, virus develops drug resistance at multiple steps resulting in treatment failure. The HIV-1 transactivator of transcription (Tat) protein engages positive transcription elongation factor b (pTEFb) complex (cycT1 and CDK9), increasing RNA pol II activity and driving viral transcriptional elongation [17]–[19]. Tat activity is enhanced by host factors such as Tat-associated histone acetylases (TAH), p300/CBP, GCN5, and P/CAF, as well as P300/CBP and GCN5 acetylate Tat at Lys 50 and 51 [20]–[23]. P/CAF acetylates Lys 28 on Tat and increases its ability to recruit pTEFb complex [20]–[22]. Thus, Tat is an important therapeutic target, having the ability to interrupt the viral life cycle. Coincidentally, no effective HIV-1 transcriptional inhibitor is yet available to complement cART. Thus, the alternative to keeping the virus in an under-expressed state until the infected cells have died is to inhibit HIV-1 transcription and subsequent viral protein synthesis, which requires new inhibitors.

**Figure 1:**
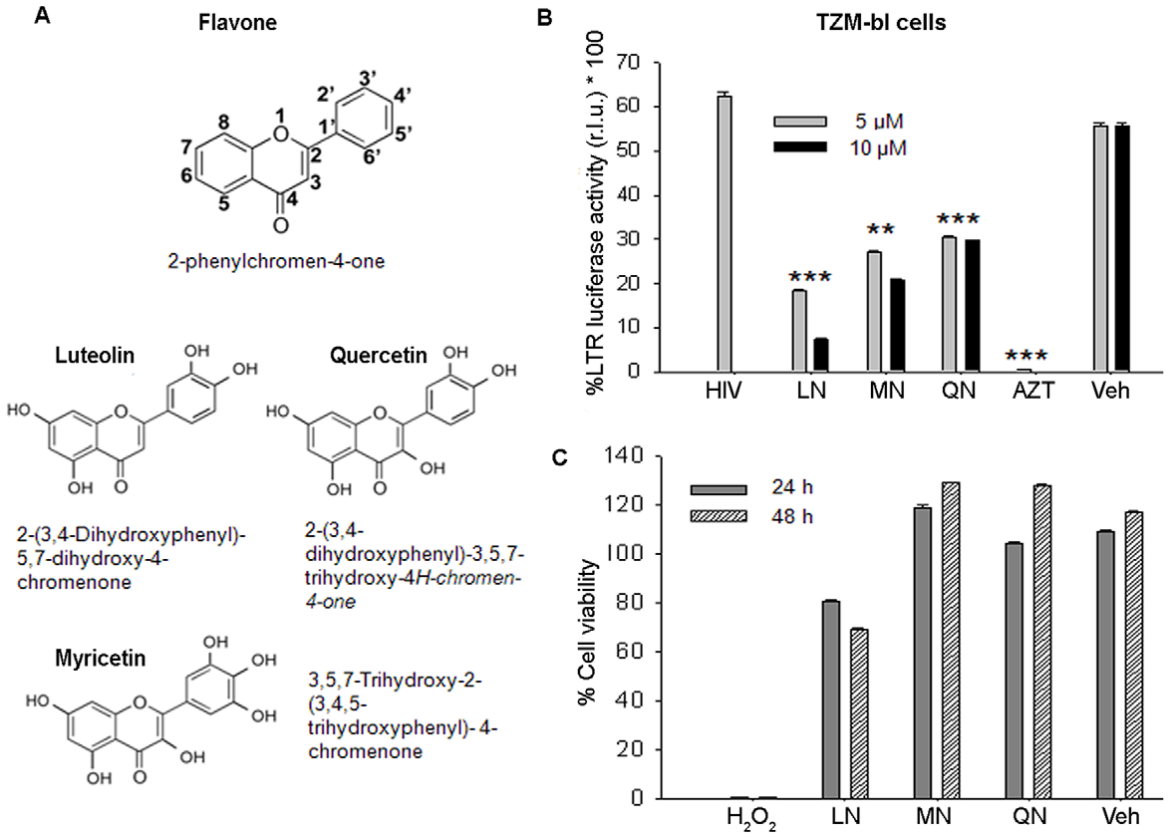
Inhibition of HIV-1 by flavonoids. (**A**) Chemical structures of flavone and its derivative flavonoids. (**B**) HIV-1 infection in LTR-luciferase TZM-bl reporter cells after 48 h treatment with luteolin (LN), myricetin (MN), or quercetin (QN), using DMSO as vehicle (Veh) or AZT (positive control). Results were plotted as mean ± SEM of duplicate readings normalized with cell control. (**C**) TZM-bl cell viability was assessed using WST-8 assay (Dojindo) after treatment for 24 and 48 h with flavonoids (10 µM); in parallel, H_2_O_2_ was used as positive control. Results are shown as percent viability relative to cell control (n = 3). **p<0.01, ***p<0.001.

Flavones, a class of flavonoids containing a characteristic 2-phenylchromene-4-one ring structure (Fig. 1a), are found in many herbs. They have shown therapeutic value, including antiviral and anti-inflammatory properties [24]–[27]. Luteolin (2-(3,4-Dihydroxyphenyl)- 5,7-dihydroxy-4-chromenone), myricetin, and quercetin, which are structurally related flavones (Fig. 1a), act as anti-oxidants and free-radical scavengers, dramatically reducing inflammatory responses [24]–[25], [28]–[32]. Their anti-oxidant property is related to the number and position of their hydroxyl groups [33]. Luteolin occurs in parsley, artichoke leaves, celery, peppers, olive oil, rosemary, lemons, peppermint, sage, and thyme; it acts as an anti-oxidant and anti-viral agent and is now being used in clinical trials for the inhibition of neuro-inflammation [24], [29]. Luteolin also has been found to have anti-HIV-1 activity [31], [34]. Although luteolin is a promoter of carbohydrate metabolism and an immune system modulator, it has been shown to have potent anti-inflammatory activity by inhibiting nuclear factor kappa B (NF-kB) in macrophages and other immune cells [35]–[36].

In addition, luteolin was shown to be effective against SARS coronavirus in a study using recombinant HIV-1 pseudotyped with SARS CoV envelope [27]. Another study found HIV-1 protease inhibitor activity in cell-free assays, but this has not been validated in infection studies. Moreover, the precise mechanism of HIV-1 inhibition is unclear [34]. Flavonoids (quercetin, -myricetin and luteolin) are structurally closely related; they are being used as dietary supplements in the United States. They have also been investigated for their anti-HIV-1 activity and its possible mechanism of action. Although we found that these three flavonoids are nontoxic and have anti-HIV-1 activity. Luteolin was the most potent and inhibited HIV-1 infection by abrogating Tat-mediated LTR activity.

## Results and Discussion

Given the long-term persistence of intact HIV-1 reservoirs, along with the development of viral escape mutants and drug resistance, viral replication goes unchecked even by intensive therapy [9]–[14], [37]. Uninterrupted lifelong anti HIV-1 treatment has resulted in longer survival but, with the persistence of residual viral activity and immune activation [37], more than 60% of cART-treated individuals develop minor cognitive disorders [38]. In addition, anti-HIV treatment leads to immune reconstitution inflammatory syndrome (IRIS) in a substantial number of African patients [39]–[40]. Above all, development of drug resistance by HIV-1 is commonly encountered during long–term treatment. Thus, a continuing search for new therapeutic agents to target novel viral life cycle stages is needed.

**Figure 2:**
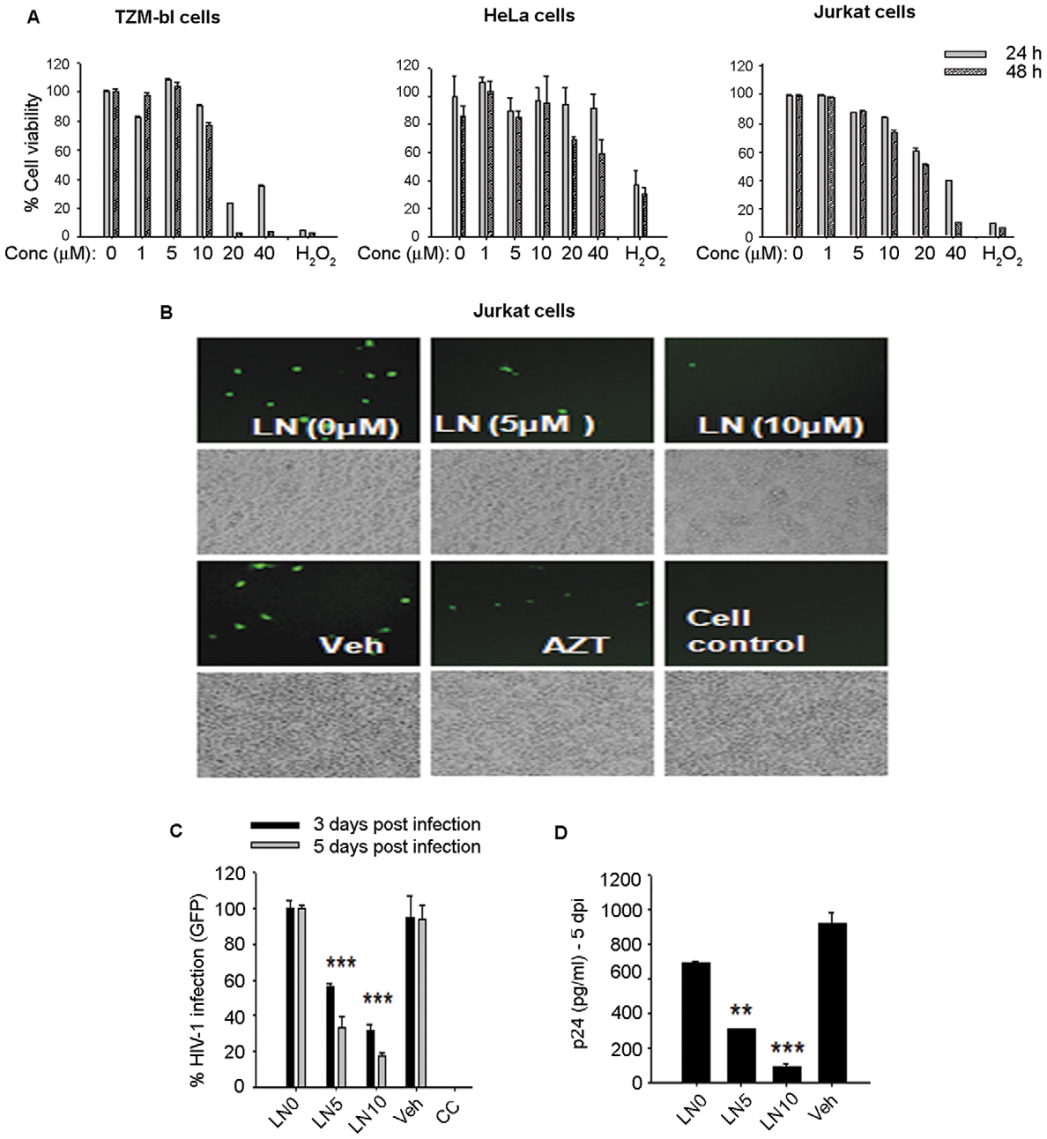
Luteolin inhibited HIV-1 infection in Jurkat cells. **A.** Viability of TZM-bl, Hela, and Jurkat cells after treatment with different concentrations of luteolin (0–40 µM) for 24–48 h as determined by WST8 -assay (n = 3). **B–D.** Jurkat cells were pretreated for 30 min with 5 µM or 10 µM luteolin (LN5 and LN10, respectively), vehicle (DMSO), or AZT (positive control) followed by HIV-1 infection for 2 h at 37°C. HIV-1 infection was monitored by (**C**) GFP expression or (**D**) virus release in supernatants as determined by p24 ELISA on the 5^th^ day post-infection. GFP quantification was done by counting 10 random low-power fields and plotted as mean ± SEM (n = 2).

The use of naturally occurring compounds such as a dietary supplement having anti-HIV-1 and anti-oxidant properties potentially provides an attractive therapy in combination with cART. We have investigated the ability of flavonoid compounds (Fig. 1A) to alter HIV-1 activity in screening assays using LTR-luciferase (TZM-bl) reporter cells and primary lymphocytes as infection models. TZM-bl cells are HeLa cells that stably express CD4 and CXCR4 receptors. They contain an integrated copy of the HIV-1 long terminal repeat (LTR) fused with luciferase and beta galactosidase genes [41]. Expression of the indicator luciferase gene is under the control of LTR via transactivator Tat protein, which is synthesized by either the viral infection or the plasmid expression vector. TZM-bl cells were treated for 30 min with luteolin, quercetin, or myricetin at 5 µM and 10 µM concentrations before HIV-1 infection. Forty-eight hours later, the infected reporter cells were monitored for LTR activity using luciferase reporter assay. Intriguingly, all of the flavonoid compounds at 5 and 10 µM concentrations showed anti-HIV-1 activity, but luteolin was the most potent (Fig. 1B). HIV-1 inhibition by luteolin was specific, as vehicle control (DMSO) had no effect on LTR activity. To rule out interference by toxicity, we monitored cell viability after flavonoid treatments, using the WST8 cell–viability assay [42] on TZM-bl cells. None of the compounds showed any cellular cytotoxicity at 10 µM concentrations (Fig. 1C). Although, luteolin showed slight toxicity in TZM-bl reporter cells, it was found, on verification on Hela and Jurkat cells (Fig. 2A) and lymphocytes, to be relatively nontoxic. The toxicity of luteolin in TZM-bl cells could result from the presence of multiple stable constructs in these cells. Moreover, earlier studies on several cell systems have reported that luteolin in concentrations up to 100 µM [43] had no adverse effects. Our data suggest that the flavonoids actively inhibited HIV-1 activity, possibly through malfunctioning of Tat activity. This could be due to blockage at any step in the viral life cycle.

In further studies, we examined the anti-HIV-1 activity of luteolin in greater detail. We tested different concentrations of luteolin (0–40 µM) on cell viability in different cell models to find a workable nontoxic concentration. Luteolin in concentrations between 5 and 10 µM, the upper limit for therapeutic agents used in screening assays, had significant antiviral activity. Thus, we used luteolin at 5 and 10 µM for all experiments (Fig. 2). We confirmed the anti-HIV-1 activity of luteolin in infection assays in lymphocytic cells and primary human lymphocytes using either wild-type (NLENG1) or VSV-pseudotyped HIV-1. In infection studies, we used HIV-1 NLENG1 containing GFP gene cloned between the envelope and nef region [44], [45]. Thus, we could monitor productive viral gene expression not only by GFP expression, using fluorescence microscopy, but by HIV-1 p24 capsid protein in the supernatants, which was detected by ELISA [42], [44].

**Figure 3:**
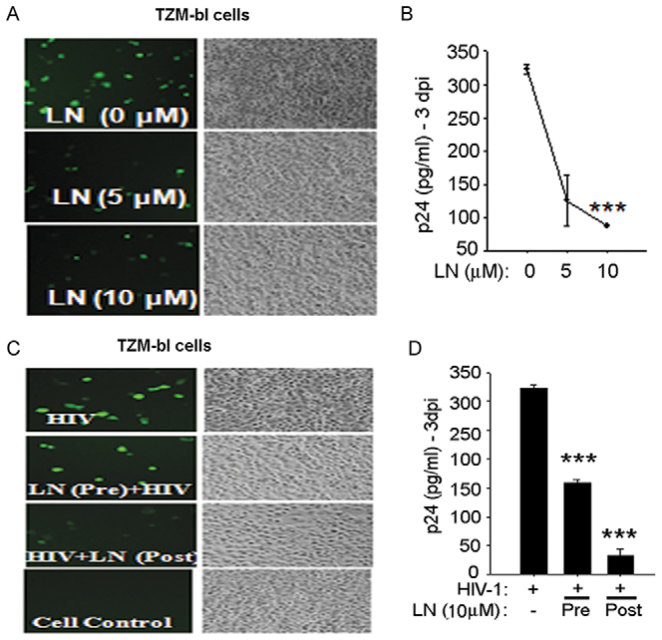
Luteolin inhibited HIV-1 infection in TZM-bl reporter cells. TZM-bl cells were pretreated for 30 min with 0, 5, and 10 µM luteolin, then infected with HIV-1 for 2 h at 37°C. HIV infection was monitored by (**A**) GFP expression or (**B**) virus released in supernatants as determined by p24 ELISA on 3^rd^ day post- infection (n = 3). **C, D**. TZM-bl cells were pre- or post-treated with luteolin (10 µM) followed by HIV-1 infection. (**C**) At 72 h post-infection, HIV-1 infection was monitored by GFP expression. (**D**) Culture supernatants were analyzed for the virus p24 antigen by ELISA. (n = 2).

Jurkat cells were pretreated with luteolin or vehicle (DMSO) for 30 min followed by HIV-1 infection, as reported earlier [42]. After infection, luteolin treatments were maintained for 3–5 days, the duration of the experiment. Luteolin treatment markedly reduced the HIV-1 infection in Jurkat cells (Fig. 2B–D) in a dose-dependent manner as shown by reductions in both the number of GFP-positive cells and viral p24 release in the supernatants on the fifth day after infection. To confirm these above findings and also rule out the cell–specific inhibition of HIV-1, we infected TZM-bl cells and obtained similar suppression of viral infection (Fig. 3A–D). Although treatment with luteolin prior to HIV-1 infection had no additional advantage on virus replication as compared with overnight treatment after HIV-1 infection (Fig. 3C, D). Instead better HIV-1 inhibition was seen with the latter, indicating that luteolin may affect the later stages of the HIV-1 life cycle rather than restricting the virus at the receptor, reverse transcription or viral DNA integration steps.

**Figure 4:**
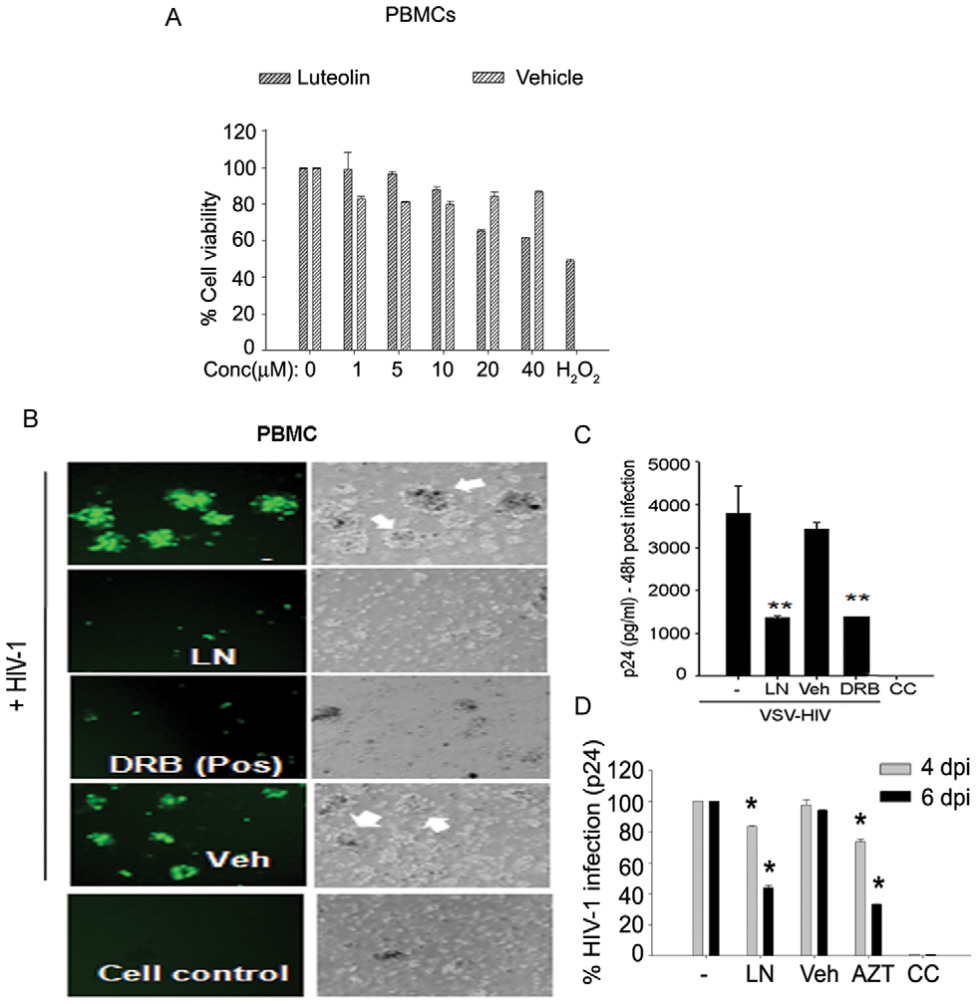
Luteolin inhibited HIV-1 infection in primary human lymphocytes. (**A**) Viability of primary human lymphocytes after treatment with different concentrations of luteolin (0–40 µM) for 24–48 h as determined by MTT assay (n = 2). (**B–D**) Luteolin inhibited HIV-1 infection in primary lymphocytes. Primary human lymphocytes were cultured in 12–well culture plates for 6 days in PHA (1%) and IL-2 (10 ng/ml), treated either with luteolin (10 µM) or vehicle, then infected with VSV-HIV-1 or wild–type HIV-1. Viral infection was monitored 2, 4, and 6 days post infection. In parallel, DRB (10 µM) was used as a positive control and DMSO as a vehicle control. (**B**) The reduction in syncytia formation is evident (white arrows) in luteolin and DRB-treated cells. (**C, D**), p24 levels in supernatants were monitored by ELISA at (**C**) 2 days after VSV-HIV infection. (** p<0.01). (**D**) 4 and 6 days after wild–type HIV-1 infection of lymphocytes (*** p<0.001).

To test this possibility, we did infection studies on primary human lymphocytes using wild type virus and additionally VSV-NLENG1 (HIV-1) was used to facilitate rapid and homogeneous receptor-independent viral infection. Before these studies, we confirmed that luteolin was non-toxic to primary cells (Fig. 4A). We treated wild-type and VSV-HIV-1 infected lymphocytes with 5 and 10 µM of luteolin until follow-up and monitored for viral infection by GFP expression and the release of p24 in culture supernatants (Fig. 4B–D). Luteolin treatment alleviated HIV-1 infection (wild–type or VSV-HIV) as shown by a reduction in GFP expression (Fig. 4B) and p24 levels in the supernatants following virus release at 2, 4, and 6 days after infection (Fig. 4C, D). In parallel, 5, 6- dichloro- 1-β -D-ribofuranosyl-1H-benzimidazole (DRB), a transcriptional inhibitor (RNA pol II inhibitor) was used as a positive control, while vehicle (DMSO) was used as a solvent control. DRB suppresses HIV-1 transcription by inhibiting RNA polymerase activity [46]–[47]. Interestingly, luteolin treatment of HIV-1 infected lymphocytes also showed inhibition in cell aggregation/syncytia similar to that produced by DRB and cell control (Fig. 4B), suggesting that viral envelope (gp120) protein expression on cell surfaces is impaired. Viral gp120 expression on infected cells is required for syncytia formation or aggregation of cells. In addition, luteolin did not affect the proliferation of lymphocytes during viral inhibition studies.

**Figure 5:**
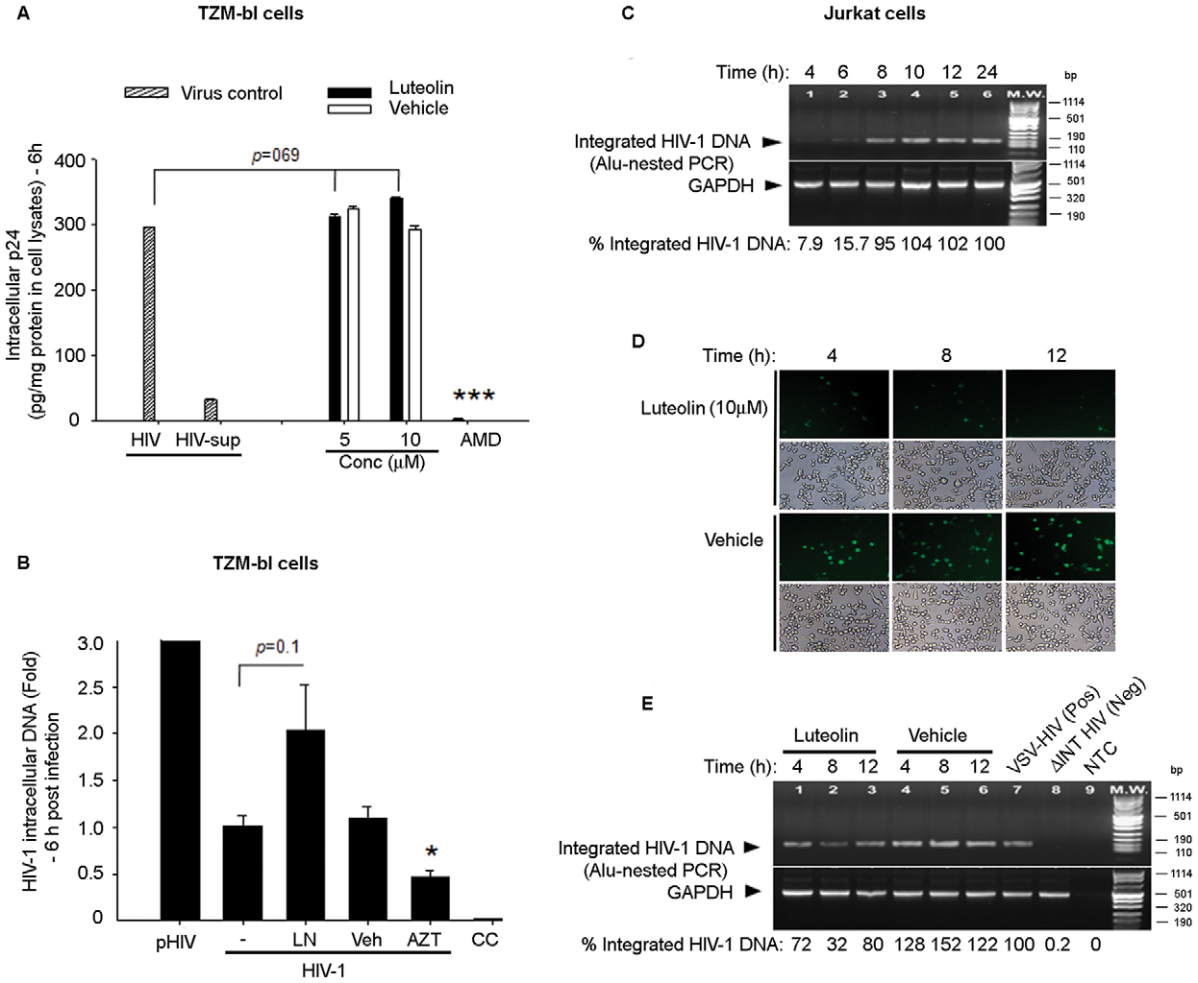
Luteolin inhibited HIV-1 independently of viral entry and reverse transcription steps. **A**. Effect of luteolin on viral entry. TZM-bl cells (6×10^5^) in six well tissue culture plates were pretreated with luteolin (5 and 10 µM) or vehicle for 1 h, then infected with HIV-1 infection (p24 = 250 ng/ml) for 2 h at 37°C. After infection, cells were briefly treated with 0.2% trypsin-EDTA and washed extensively to remove cell-membrane-bound virus particles. Six h post-infection, cells were trypsinized and lysed. p24 levels were estimated in cell lysates after normalization of protein concentrations (BCA method) and in HIV-1 infected culture supernatant (HIV-sup). The results are presented as the amount of p24 present per mg of proteins in cell lysates. **B**. TZM-bl cells (6×10^5^) in six–well tissue culture plates were pretreated with luteolin (10 µM) or DMSO for 30 min, then infected with HIV-1 NLENG1 (p24, 250 ng/ml) for 2 h. At 6 h after infection, cells were treated briefly with 0.2% trypsin and washed. Genomic DNA was harvested from HIV-infected cells. 200 ng of total DNA was used as a template for quantification of viral DNA by real-time PCR using Tat primers and normalized to GAPDH signals. In parallel, 500 ng HIV-1 proviral DNA (pHIV) was transfected as a positive control. **C**. Jurkat cells (7×10^5^) in six–well culture plates were infected with VSV-HIV-1 (p24 = 250 ng/ml) for 2 h at 37°C, washed twice, and followed up for 24 h. Cells were harvested from 0 to 24 h after infection and viral integration was monitored by Alu-LTR-PCR. **D–E**. Jurkat cells were infected with VSV-HIV-1 and treated with luteolin (10 µM) or DMSO at 4, 8, and 12 h after infection. The levels of viral infection were monitored by the amount of GFP expression in luteolin- and vehicle-treated HIV-1 infected cells (**D**). Viral integration was analyzed 24 h post-infection by Alu-LTR PCR (**E**). VSV-HIV-1 was used as positive control; HIV-1 NL4-3 mutant (D64A) defective in viral DNA integration function (ΔINT HIV) was used as negative control (n = 4).

Our results thus far indicated that the mechanism of luteolin-mediated HIV-1 suppression is independent of viral entry receptors. To corroborate the effect of luteolin on wild–type virus, TZM-bl reporter cells were pretreated with luteolin or DMSO for 30 min, then infected with HIV-1 (p24 = 250 ng/ml). We assessed the effect of luteolin on virus entry after 6 h of infection by the accumulation of intracellular viral capsid (p24), which was quantified by ELISA in HIV-1 —infected cell lysates. As a positive control for entry inhibition, we pretreated TZM-bl cells with a CXCR4 blocker, AMD 3100, before infecting them with HIV-1. Luteolin (5–10 µM) pretreatment did not have a significant effect on wild–type HIV-1 entry as compared to that in untreated infected cultures (Fig. 5A). To rule out the possibility of contamination from cell-membrane-bound virus particles in the cell lysates, we tested p24 levels in HIV-1-infected cell supernatants (HIV-sup) at 6 h after infection and found barely detectable levels (Fig. 5A).

The preceding results showed that luteolin inhibited HIV-1 infection independent of viral entry. We then investigated whether luteolin had any effect on viral reverse transcription. After infecting TZM-bl cells with HIV-1 for 2 h and treating them with luteolin, we monitored the levels of reverse-transcribed intracellular HIV-1 DNA at 6 h after infection, using real-time PCR [48]. In this situation, unlike that when we used AZT, a reverse transcriptase inhibitor (positive control), treating HIV-1 infected TZM-bl cells with luteolin did not significantly alter the amount of reverse transcribed HIV-1 DNA (Fig. 5B). This suggested that luteolin was unable to annihilate viral reverse transcription.

We then investigated the effect of luteolin on viral integration, first testing viral integration kinetics in VSV-pseudotyped HIV-1-infected Jurkat cells from 0 to 24 h using semiquantitative Alu-integration PCR, with GAPDH as an internal control [44]. The minimum PCR amplification cycles were optimized to obtain the amplification product in linear log phase. Densitometric analysis of amplified PCR products showed that viral integration was rapid in Jurkat cells, starting within just 4 h after infection (Fig. 5C). This is consistent with earlier findings on VSV-HIV-1 [49]. However, our results suggest that the peak integration levels are reached by 8–10 h after infection, slightly longer than in an earlier study [49]. These differences could be due to use of different HIV-1 strain in the above study.

To test the effect of luteolin on viral integration, we infected Jurkat cells with VSV-pseudotyped HIV-1, then treated the cells with either luteolin or vehicle at times ranging from 4 to 12 h. After 24 h, viral infection was monitored by GFP expression and viral integration, using Alu-LTR PCR as described (Fig. 5D, E). In parallel, we used integrase-defective mutant HIV-1 (D64A) as a control [50], [51]. As compared to vehicle controls, luteolin-mediated suppression of HIV-1 expression was evident irrespective of the time after infection when treatment was initiated (4–12 h) (Fig. 5D). The weak suppression in integration signal as compared to that in untreated infected cells (Fig. 5E) was in contrast to a previous report showing exclusive effect on viral integration [31]. Intriguingly, the suppression of HIV-1 infection in cells treated with luteolin at 12 h post-infection suggests that luteolin has an inhibitory effect on the HIV-1 life cycle mainly if not exclusively at the postintegration stage.

**Figure 6:**
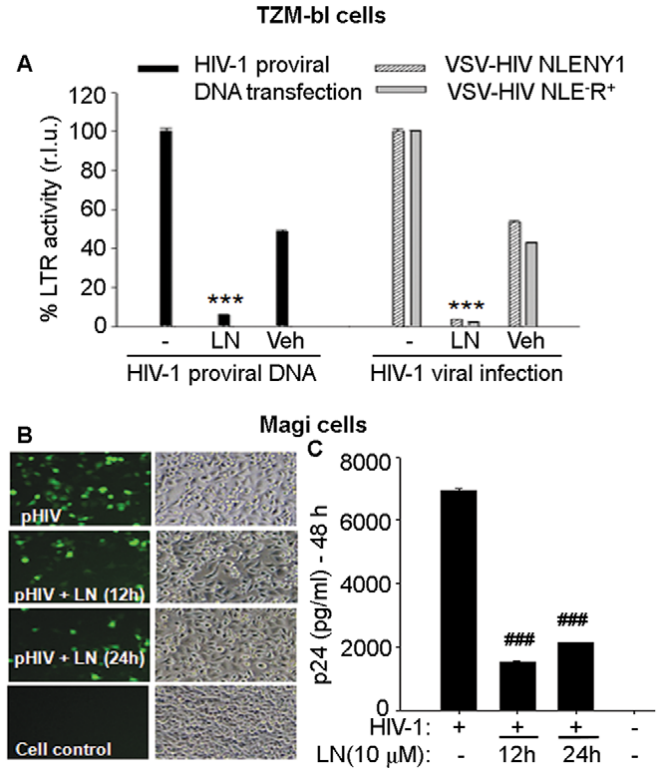
Luteolin inhibited HIV-1 gene expression independently of viral DNA integration. **A**. TZM-bl reporter cells in 12 well culture plates were transfected with HIV-1 plasmid DNA vector expressing GFP, then treated with luteolin (10 µM) or DMSO (Veh). In parallel, TZM-bl cells were infected with VSV-HIV NLENG1 or NLR^+^E^−^ for 2 h, then treated with luteolin (10 µM) or DMSO for the duration of follow up. At 48 h post-transfection or infection, cells were lysed and assayed for luciferase activity (n = 2). **B–C**. Two hours after Magi cells were transfected with pHIV NLENG1 (150 ng), luteolin (10 µM) was added to them. After 6 h, transfection medium was replaced with fresh medium containing luteolin for 12 or 24 h. At 48 h post-transfection, cells were monitored for GFP expression. Representative pictures are shown (**B**). Cell supernatants were collected to measure p24 levels (**Cs**) (n = 2). *** p<0.001, ### p<0.005.

Given that integration was already complete within 8–10 h post-infection (Fig. 5C), we further explored the effect of luteolin on post-integration steps of the HIV-1 life cycle. To investigate HIV-1 replication independent of viral DNA integration, we transfected TZM-bl cells with HIV-1 DNA expression vector. After treating the cells with luteolin or vehicle control (DMSO), we monitored viral gene expression using the LTR-luciferase assay. In this system, Tat expression from HIV-1 DNA drives LTR-luciferase expression, which was quantified luminometrically. Luteolin treatment markedly inhibited HIV-1 gene expression (or at least Tat expression) as compared to that in control TZM-bl reporter cells (Fig. 6A). Given that HIV-1 plasmid DNA expression vector replicates independently of viral DNA integration, it is evident that luteolin inhibited either Tat expression or Tat function at the LTR level, but not HIV-1 DNA integration.

To validate the HIV-1 plasmid DNA expression results, we did infection studies in which VSV-pseudotyped HIV-1 wild-type viruses or envelope-deficient HIV-1 viruses were used on TZM-bl reporter cells. As compared to controls, luteolin-treated VSV-HIV-1-infected reporter cells profoundly suppressed the Tat-regulated luciferase signal similar to that in HIV-1 plasmid DNA transfection (Fig. 6A), suggesting aborted viral replication. DMSO controls also showed some suppression in luciferase signal in both HIV-1 DNA transfected and VSV-virus infected TZM-bl cells. One possibility is that DMSO affects luciferase expression either at the mRNA level or via CMV promoter. However, we confirmed our HIV-1 inhibition results on Magi cells (CD4/CCR5+ve HeLa cells) using GFP and viral p24 as markers. We transfected these cells with recombinant HIV-1 plasmid DNA vector expressing GFP (NLENG1), then treated them with luteolin for 12–24 h. At 48 h after transfection, we monitored GFP expression and viral p24 antigen in the supernatants, finding that luteolin treatment suppressed both (Fig. 6B, C), which suggested inhibition at the post-HIV-1 DNA integration step. We are convinced that small inhibition in DMSO-treated VSV-HIV-1 infected cultures or HIV-1 DNA transfected reporter cells was made possible by the effect on viral (VSV) endocytosis. However, we do not underestimate the true effect of luteolin, which was corroborated by inhibition of wild-type infection, HIV-1 DNA-mediated LTR transactivation, and post-integration viral state without any inhibitory effect of DMSO. Antiviral activity of luteolin is clearly shown in wild–type HIV-1 infection studies wherein DMSO did not confer any antiviral activity. These results ruled out any additive effect of DMSO with luteolin on HIV inhibition.

**Figure 7:**
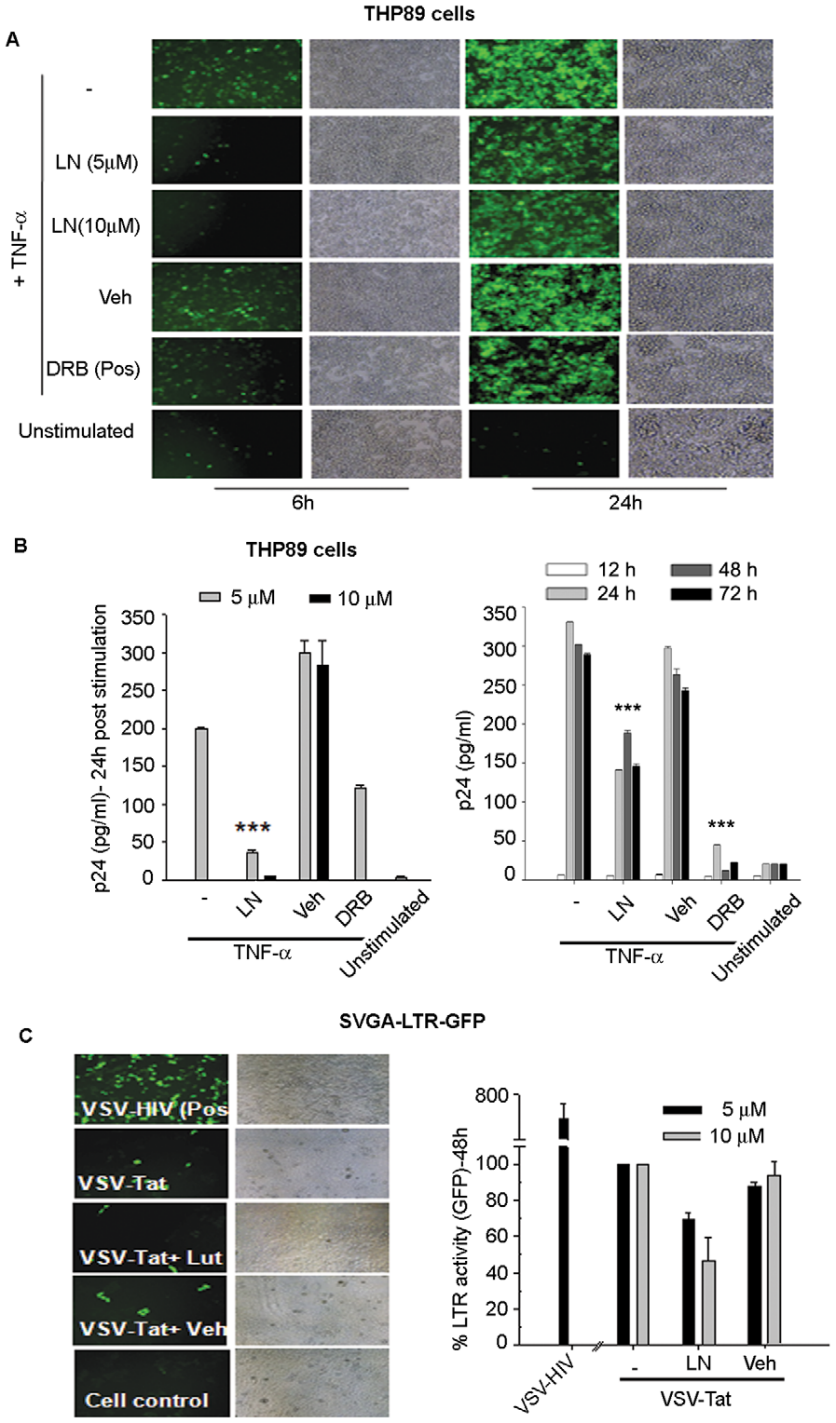
Luteolin inhibited reactivation of latent HIV-1 infection. (**A, B**) One million latently HIV-infected THP89 cells seeded per well in 12 well culture plates were pretreated overnight with luteolin (5 or 10 µM) or DMSO, then stimulated with TNF-α (10 ng/ml) and monitored by (**A**) GFP expression and (**B**) virus production by p24 ELISA. Similarly, 10 µM luteolin treatments were given from 12 h to 72 h post -TNF-α stimulation. Virus production was monitored by p24 ELISA. DRB (10 µM) was used in parallel as a positive control (n = 3). (**C**) Luteolin inhibited transactivation of integrated HIV- LTR. SVGA-LTR-GFP reporter cells were transduced with VSV-Tat viral particles for 2 h, then treated with 10 µM luteolin or vehicle and, after 24 h, monitored for GFP expression.

Thus far, luteolin-mediated suppression of the LTR-luciferase activity has been demonstrated after HIV-1 plasmid DNA transfection or HIV-1 infection, suggesting that luteolin may confer anti-viral activity at the functional level of Tat-LTR function, Tat expression and its sub-cellular localization or downstream viral protease. To further examine luteolin-mediated inhibition of HIV-1 at post–DNA integration stages, we did experiments on latently HIV-1 infected THP89 cells, in which the HIV-1 genome contains the GFP gene. THP89 cells contain HIV-1 provirus in a latent state without any viral gene expression including GFP [44]. On induction by TNF-α (NF-κB activation) or Trichostatin A (histone deacetylase inhibitor), however, viral reactivation is initiated; cells start expressing GFP and release the virus extracellularly [32], [45], [48]. After THP89 cells had been pretreated with luteolin or DMSO for 18 h, viral reactivation was initiated using TNF-α (10 ng/ml). TNF-α- reactivated latent HIV-1 infection was attenuated by treatment with luteolin (10 µM), demonstrating reduction in GFP expression and released virus (p24) in 24, 48, and 72–h induction periods (Fig. 7A–B). This indicates that luteolin has an effect on post-viral DNA integration stages; that is viral transcription or translation or Tat functional activity. When Tat functional activity is impaired in a TNF-α —reactivated latent HIV-1 model, GFP expression, including viral proteins that are dependent on Tat function, will eventually be impaired, so that p24 activity is reduced in infected culture supernatants.

**Figure 8:**
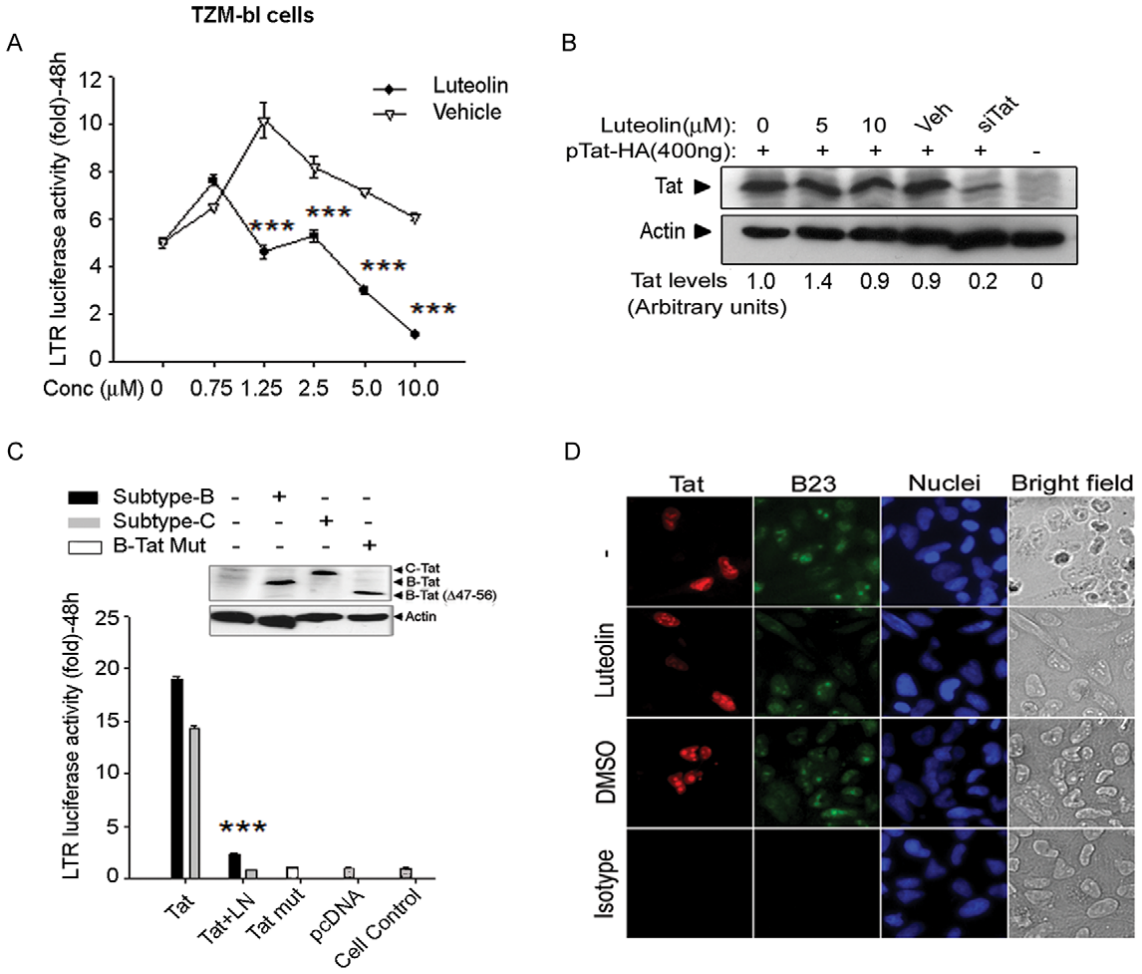
Luteolin inhibited clade B and –C Tat–mediated LTR transactivation in TZM-bl reporter cells. (**A**) TZM-bl reporter cells were transfected with Tat expression vector (pcDNA-Tat) and treated after 4 h with different concentrations of either luteolin (0–10 µM) or vehicle and monitored for luciferase activity. (**B**) TZM-bl cells were transfected with pIRES2-EGFP-Tat-HA (400 ng) and, 24 h later, treated with 0-, 5- and 10- µM luteolin. A Tat-specific siRNA cocktail of 3 siRNAs (300 nM) was co-transfected with Tat expression vector as a positive control. 48 h post-transfection, cells were harvested for Western blot using anti-HA and anti-β actin antibody. (**C**) TZM-bl cells transfected with HIV-1 subtype-B or -C Tat expression vectors (pcDNA-Tat) were treated with luteolin (10 µM) at 4 h after transfection. In parallel, mutant Tat-47 (Δ 47–56 aa) was used as a negative control. LTR luciferase activity was assessed at 48 h after transfection. Protein levels expressed from Tat expression vectors were monitored by Western blot with anti-HA and anti-β-actin antibody. *** p<0.001. (**D**) Effect of luteolin treatment on subcellular localization of Tat protein in HeLa cells. Immunostaining showing subcellular localization of Tat protein in HeLa cells after treatment with luteolin (10 µM), DMSO as a vehicle control (DMSO), or untreated (-). IgG was used as isotype antibody control (Isotype). Cells were immunostained for Tat (red), B23/nucleophosmin (green), and nuclei (blue), images were captured at 20× with a Nikon fluorescent microscope.

Taken together, our multiple lines of evidences indicated that luteolin confers its anti-HIV-1 activity by inhibiting HIV-1 transcription, translation, or post–translational processing (protease). However, given our finding that GFP expression was reduced in both TNF-α reactivated latent HIV-1 infection and acute viral infection; the data point toward transcriptional inhibition or translation (Figs. 2, 3, 4, 5, 6, and 7), which was not expected from crippled viral protease. GFP is cloned between env and nef genes [44]–[45] and its expression is independent of viral protease activity, but dependent on Tat for transcription. To validate the infection data, we examined Tat function, using only Tat expression vectors to rule out other viral proteins. To mimic HIV-1 expressed Tat, we packaged lentiviral-vector- expressing Tat protein pseudotyped with VSV envelope (VSV-Tat). We used VSV-Tat viral particles for transduction of SVGA-LTR-GFP reporter cells expressing EGFP under the control of HIV-1 LTR [52]–[53]. In parallel, VSV-pseudotyped HIV-1 was used as a positive control. Luteolin treatment following transduction with VSV-Tat virus particles led to suppression of LTR-mediated GFP expression (Fig. 7C), suggesting compromise in Tat function at either the protein expression level or the LTR-transactivation level. We corroborated these results using TZM-bl reporter cells. Transfection of pcDNA-Tat expression vector wherein Tat is under the control of CMV promoter, followed by treatment with different concentrations of luteolin (0–10 µM), resulted in dose-dependent suppression of LTR-luciferase expression, which did not occur in vehicle-treated or untreated cultures (Fig. 8A).

We further investigated whether luteolin affects Tat expression levels by transfecting TZM-bl cells with Tat expression vector (pcDNA-Tat), and treating them with either luteolin or vehicle. Luteolin treatment had no affect on Tat protein levels as analyzed by Western blotting (Fig. 8B), indicating that LTR transactivation (Fig. 8A, C) is restricted at the Tat functional level. In parallel, we co-transfected Tat siRNA with Tat expression vector (pcDNA-Tat) as a positive control, observing suppression in Tat expression, but not in vehicle controls (Fig. 8B).

The variation in transcriptional activity of HIV-1 Tat protein among HIV-1 subtypes has been predominately attributed to sequence variability in its N-terminal activation domain (1–48 amino acids), especially the cysteine–rich region, which binds Zn^2+^ cation and engages cyclin T1 [54]. Tat from HIV-1 subtype-C and E (prevalent in Asia and Africa) is reported to exhibit higher transactivation and TAR RNA binding capacity than does HIV-1 subtype-B (prevalent in North America and Europe) [54]–[56]. Hence, we investigated whether luteolin-mediated suppression of Tat-mediated LTR-transactivation can be extended to different HIV-1 subtypes. On transfection of clade-B and C Tat expression vectors (pcDNA-Tat vectors) abundant expression of Tat proteins was found in TZM-bl cells (Fig. 8C, inset), with levels of LTR-transactivation similar to those in compared to controls observed in Western blots (Fig. 8C). In further experiments, luteolin treatment profoundly inhibited LTR-mediated luciferase expression in both clade-B and -C Tat transfected cells (Fig. 8C). In parallel, transcriptionally inactive Tat-47 mutant (Δ48–56) deleted in nuclear localization signal was used as negative control, demonstrating only basal luciferase activity in both Tat-47- and empty vector (pcDNA)- transfected TZM-bl cells.

**Figure 9:**
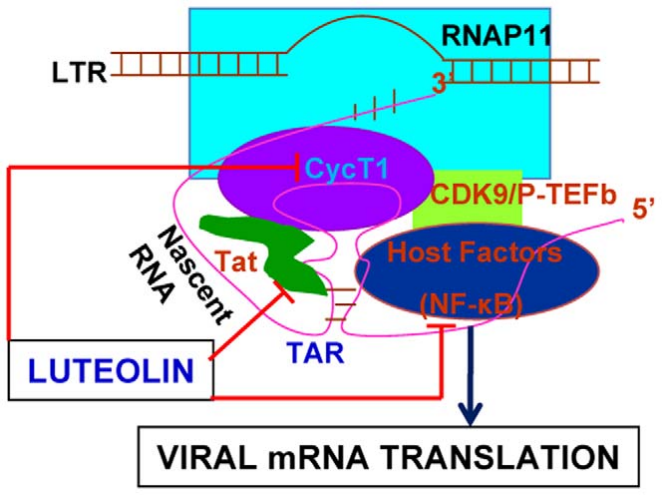
Proposed schematic representation of anti-HIV activity of luteolin. After HIV-1 DNA integration into host genome, viral genes are expressed under the control of the HIV-1 long terminal repeat (LTR) as a promoter with the help of viral regulatory protein Tat, which binds with TAR RNA element in the 5′ end of LTR. Luteolin may abrogate Tat-mediated LTR transactivation activity by interfering with pTEF-b binding with LTR or abolish Tat binding; it also may prevent NF-κB activation or inhibition of host factors involved in transcription or inhibition of viral mRNA translation.

Given that Tat is a bona fide nuclear/nucleolar protein [52], [57]–[59], we investigated whether luteolin affects sub-localization and thus cripples HIV-1 LTR-activity. We transfected HeLa cells using Tat-HA vector and 6 h after transfection, then treated them with luteolin or DMSO or left them untreated. At 24 h after transfection, cells were immunostained for Tat, nucleophosmin (nucleolar protein), and nuclei, then examined for Tat localization. Tat sub-cellular (nuclear/nucleolar) localization was unaffected by luteolin, as in DMSO- or mock- treated cells (Fig. 8D). This indicates that viral transactivator protein function is impaired at the LTR-functional level, including either Tat binding to TAR RNA or cyclin T1/CDK9 or inhibition of critical factors, including NF-κB, that are involved in HIV transcription (Fig. 9).

These observations confirm that luteolin confers its anti-HIV-1 activity at the Tat-LTR transactivation level (transcription step) after the viral integration step. However, further investigations of the action of luteolin are needed to decipher the detailed mechanism of Tat-mediated LTR inhibition. Currently, we are investigating the interactions of Tat with HIV-1 LTR and host transcription factors, as well as post–translational modification on Tat in the presence of luteolin. Several compounds, such as Quinolines, as well as stilbene- and purine-derivatives have been reported to show anti-HIV activity, inhibiting Tat-TAR interactions [60]–[64]. Other compounds, such as flavipiridol, rescovtine, and 9-amino acridine, targeted CDK9, a component of pTEFb complex [65]–[67]. Recently, a coumarin derivative has been shown to potently inhibit HIV Tat function in nano–molar concentrations by repressing p300 and PI3K/AKT [68]. This supports the possibility that luteolin leads to the development of potent derivatives to inhibit HIV in nano–molar concentrations. Therefore, several other active derivatives of luteolin shown to have more potent antioxidant and anti-inflammatory properties need to be investigated for anti-HIV-1 activity. Intriguingly, quercetin, a luteolin analogue, has been shown to repress proinflammatory miRNA-155 in macrophages in –vivo [43], it would be interesting to determine whether luteolin has the same effect. One caveat is that luteolin may complement combination anti-HIV therapy by preventing synthesis of viral proteins available for protease steps. This would enhance the potency of protease inhibitors, eventually leading to reduced virus assembly and release.

In conclusion, our results on reporter cells and primary lymphocytes indicate that luteolin cripples HIV-1 Tat function and may be the critical component in anti-retroviral combination therapy for HIV-1 infection. In addition, luteolin crosses the blood-brain barrier and has been shown to confer neuroprotective effects via inhibition of neuroinflammation [28], [69]–[71]. However, the concentrations of luteolin that we found to be inhibitory in our study are in the upper therapeutic ranges. Further investigation is needed to determine whether these concentrations are achieved in vivo. Further investigations in–vivo and significance of luteolin with anti-HIV drugs whether it can act in synergism with lower concentrations, are the future goals. However, we have laid the foundation for development of luteolin derivatives that, in nanomolar ranges, may which could inhibit HIV-1. Currently, we are investigating the ability of 500 flavonoid derivatives to inhibit HIV in nano molar ranges.

## Materials and Methods

### Primary cells, cell lines, and reagents

Human peripheral blood mononuclear cells (PBMCs) were separated from whole blood (New York blood bank) using Histopaque (Sigma, St., MO), washed three times with phosphate-buffered saline (PBS), and cultured in 10% FBS containing RPMI as previously described [42]. TZM-bl (CD4/CXCR4/CCR5+ve HeLa cells) and Jurkat cells were obtained through AIDS Reagent and Reference Program, NIH. TZM-bl, SVGA-LTR-GFP cells [52] were grown in DMEM while Jurkat cells were grown in RPMI with 2 mM L-glutamine and RPMI (Gibco-BRL), each supplemented with 10% FBS, 1% penicillin, streptomycin, and amphotericin B (GibcoBRL). We obtained all chemical reagents from Sigma (St. Louis, MO). Stocks for luteolin, quercetin, and myricetin were prepared in dimethyl sulphoxide (DMSO). All flavonoids used in the study were of more than 98% purity. Rabbit polyclonal anti-HA antibodies were purchased from Santa Cruz Biotechnology, mouse anti-B23 (nucleophosmin) antibody from Abcam, Alexa 568 conjugated goat anti-rabbit IgG, and Alexa 488 conjugated goat anti-mouse IgG from Invitrogen. We purchased mouse monoclonal anti β-actin antibody from Sigma.

### Viral constructs and plasmids

We obtained HIV-1 NL 4-3.HSA.R^+^E^−^ [72], subtype C Tat [73], and VSV-G expression plasmids [74] through the National Institutes of Health (NIH) AIDS Reagent and Reference Program. The use of HIV-1 NLENG1 has been described [44], as the use of NL4-3 integrase mutant (D64A) [50]. Full-length subtype B Tat (101 amino acids) was subcloned from pcDNA-Tat [53] into HIV-1 derived lentiviral vector pLVX (Clontech) under CMV promoter and packaged with a VSV envelope as described earlier [48]. HA tag was inserted at the C-terminus of full-length subtype B and subtype C Tat, along with EcoRI and XhoI restriction sites by PCR amplification. Amplicon was cloned into pcDNA3.0 vector. Similarly, subtype B Tat with HA tag was subcloned into bicistronic pIRES2-EGFP vector (Clontech). Functionally inactive subtype B mutant was created by deletion of nuclear localization signal (amino acids 47–56) and was cloned with HA tag in pcDNA3.0 vector at EcoRI and XhoI sites. The inserts were confirmed by double–strand DNA sequencing. The HIV-1 long terminal repeat (LTR)-driven GFP construct was created by replacing CMV promoter in the pEGFP-N1 vector (Clontech) with LTR promoter at SalI and SmaI, then stably transfected into SVGA cells [53].

### Virus packaging, pseudotyping, and HIV-1 infection assay

HIV-1 full length DNAs or lentiviral vector pLVX expressing Tat were packaged in 293T cells as described previously [42]. Briefly, we transfected 17 µg of HIV or lentiviral expression vector DNA per 100 mm culture dish (BD Falcon) using Lipofectamine 2000 (Invitrogen). Similarly, we pseudotyped HIV-1 NLENG1 or HIV-1 pNL4-3.HSA.R^+^.E^−^ proviral DNA (17 µg) with VSV-G envelope using a 4.0 µg VSV-G construct. To pseudotype lentiviral vector, cells were transfected with 10.0 µg pLVX-Tat or pLVX with 3.0 µg pCMV-Tat, 8.0 µg pGag-Pol, 4.0 µg pCMV-Rev, 2.5 µg pVpr, and 4.0 µg pVSV-G using Lipofectamine 2000 [42]. The supernatants containing viral particles were harvested 72 h after transfection and centrifuged at 300 g for 10 min to remove cellular debris. Further, viral stocks were treated with 5 IU/ml of RNase-free DNase for 15 min at room temperature and membrane–filtered. Stocks were filtered and stored as 1.0 ml aliquots at −80°C. Viral titers were determined by p24 ELISA (Zeptometrix, Buffalo, NY) or by transducing SVGA-LTR-GFP reporter cells.

HIV-1 infection in either TZM-bl or Jurkat cells was done in 12-well tissue culture plates (Falcon) with HIV-1 NLENG1 (250 ng/ml p24 antigen) for 2 h at 37°C. After infection, cells were washed twice with optiMEM and replaced with complete medium containing 10% FBS. On the next day, medium was replaced once to remove input-adsorbed virus particles released during the 24-h period. We collected supernatants on the third and fifth days after infection. p24 antigen was monitored in HIV-1 infected culture supernatants by quantitative ELISA (Zeptometrix). GFP expression was monitored and the images captured by a digital camera in a fluorescent microscope (Nikon). Primary human lymphocytes were cultured from PBMCs and stimulated with 1% PHA and IL-2 (10 ng/ml) [42], then infected with wild–type HIV (NLENY1) or VSV-HIV-1 for 2 h at 37°C, then washed. Productive virus infection was monitored at 2, 4, and 6 days after infection by p24 ELISA.

### Western blotting and immunofluorescence

TZM-bl cells were transfected with pIRES2-EGFP-Tat-HA and, on the next day, treated them with luteolin or DMSO. Cells were harvested 48 h after transfection and lysed in RIPA buffer (Sigma) containing a protease inhibitor cocktail (Pierce); 30 µg of total protein from each sample was then analyzed on 12% SDS-PAGE. Protein bands were transferred to polyvinylidene difluoride (PVDF) membranes (BioRad) [48]. We blocked the membranes with blocking buffer (5% nonfat dry milk, 0.1% Tween 20 in PBS) for 1 h at room temperature. Overnight, the membranes were probed at 4°C with rabbit polyclonal antibody against HA peptide (1∶1000, Santa Cruz Biotechnology) and, as an internal control, mouse monoclonal antibody against beta actin (1∶3000 dilution, Sigma). After three washes with 0.1% Tween 20 in PBS, the membranes were incubated with anti-rabbit IgG secondary antibody conjugated to horseradish peroxidase (1∶3,000, BioRad) in blocking buffer for 1 h at room temperature. We washed the membranes three times with 0.1% Tween 20 in PBS and developed them using a chemiluminescence detection kit ECL (GE Healthcare). Tat levels were quantified by densitometric analysis using Image J software (NIH). Normalization of samples was done at two levels using equal protein concentrations for loading followed by actin levels.

Immunofluorescence staining was done as described previously [44]. HeLa cells seeded in slide flaskets (Nunc, Denmark) were transfected with 500 ng of pcDNA-Tat-HA expression vector using Lipofectamine 2000. At 24 h after transfection, cells were fixed with 2% paraformaldehyde for 15 min at 25°C. The cells were washed twice with PBS and permeabilized by Triton X-100 (0.2%; v/v) in PBS for 11 min. Slides were overlaid with primary antibodies against HA (rabbit) and B23/nucleophosmin (monoclonal) as a nuclear maker, and each antibody diluted to 1∶300. Slides were kept overnight at 4°C. In parallel, IgG1 antibody was used as an isotype antibody control. The slides were washed three times with PBS and overlaid with alexa-568 conjugated goat-anti-rabbit (1∶500) and alexa-488 conjugated goat-anti-mouse (1∶500), then incubated at 25°C for 35 min. After two washings, nuclei were stained using Hoechst (1 µg/ml) for 5 min. Slides were mounted in fluoromount (Sigma), an aqueous gel mounting medium. Slides were examined with Nikon E600 fluorescence microscope and images acquired at 20× magnification.

### HIV-LTR reporter assay

We infected TZM-bl cells with HIV-1 (p24 = 250 ng/ml) or transfected them with Tat expression vector using Lipofectamine 2000 (Invitrogen) [53] either in 96-well or 12-well plates, then treated the cells with luteolin or vehicle control. The DNA concentrations in transfection were normalized using empty control vector (pcDNA). At 48 h after treatment, we lysed cells using reporter lysis buffer (Promega), then put them through one freezing-thawing cycle. 30 µl of cell lysates was used to monitor luciferase activity in a 100-µl reaction using ready-glow firefly luciferase substrate (Promega). We quantified the luminescence in optilux black plates (Falcon) using a multi-mode microplate reader (BioTek instruments), then calculated the percent of LTR luciferase activity as relative luciferase units normalized to cell control.

### Real-time PCR and Alu-HIV-1 integration PCR

To detect viral DNA synthesis after reverse transcription in HIV-1 infection studies, we did real-time PCR as described earlier [48]. Briefly, we harvested DNA from the infected cells using DNAzol (Invitrogen) [48]. The cells were removed by trypsinization and washed twice with PBS, after which we added 1.0 ml DNAzol to cell pellets. DNA was precipitated by adding 500 µl ethanol, removed by spooling with a pipette tip, and washed once with 70% ethanol. DNA was dissolved in 8 mM NaOH (pH 8.0) and spectrophotometrically quantified. The amount of viral DNA in the samples was quantified by real-time PCR [75] using Tat gene-specific primers. The following primer sets were used:

Tat forward: 5′-GAAGCATCCAGGAAGTCAGCC-3′

Tat reverse: 5′-ACAAACTTGGCAATGAAAGCAACAC-3′

GAPDH forward: 5′-CATCAGCAATGCCTCCTGCACC-3′

GAPDH reverse: 5′-GTGCTCAGTGTAGCCCAGGATG-3′.

Briefly, 200 ng of genomic DNA was used in 30-µl PCR reaction mix in triplicate with 10 pmol of each primer and 2× sybr green (SA Biosciences). The cycle program for amplification was 95°C/3 min followed by 45 cycles of 95°C/20 sec, 60°C/20 sec and 72°C/20 sec. The reactions were run on a CFX96 real-time PCR system (Bio-RAD). Data were collected and analyzed using Bio-RAD CFX Manager Software v 1.1. Ct values were calculated for each gene and normalized relative to GAPDH expression. Results were presented as mean ± SEM of two separate experiments. Fold expression from untreated controls was calculated by the 2^−ΔΔCt^ method [75].

To examine HIV-1 DNA integration, semiquantitative nested Alu-HIV-1 integration PCR was done as described previously [44], [76], but with slight modifications. The following primers were used for the first round of amplification:

INT-1: 5′- TGCTGGGATTACAGG GCGTGAG-3′

INT-2: 5′-TAGACCAGATC- TGAGCCTGGGA-3′.

The primers for second round were;

INT-N1: 5′-GGCTAACTAGGGAAC-CCACTG-3′

INT-N9, 5′-CTGCTAGAGATTTTCCACACTGAC-3′.

We used 200 ng of genomic DNA as a template for amplification with the first set of Alu-HIV-1 PCR primers in a 50-µl PCR mix. Amplification cycles were 96°C/3 min followed by 16 cycles of 96°C/45 sec, 60°C/15 sec, and 72°C/50 sec. In the nested step, 1.0 µl of the first PCR product was used as a template in a 50-µl reaction volume in duplicate and was amplified for 25 cycles using a similar PCR protocol as noted earlier. For all sample sets, GAPDH was amplified as an internal control from 200 ng genomic DNA as a template using the cycle program as 96°C/3 min followed by 25 cycles of denaturation at 96°C/45 sec, 57°C/15 sec, and 69°C/45 sec. The amplified PCR products were separated and visualized on 2% agarose gel. For quantification, densitometric analysis was done using image J software (NIH); GAPDH was used for normalization. The percent of integrated HIV-1 DNA was calculated relative to positive control (24 h after infection with VSV-HIV-1) as given below:
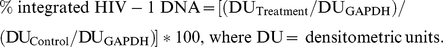



### Cytotoxicity assay

TZM-bl or HeLa cells were seeded in 96-well plates at a density of 2.5×10^4^. Jurkat cells were seeded at a density of 5.0×10^4^ per well. We cultured the cells overnight and, next day, treated the cells with luteolin or control in triplicate. After 24 or 48 h, we measured the viability of cells after removing the culture medium and adding 100 µl PBS containing 10 µl of cell counting kit-8 (CCK-8) reagent (Dojindo Molecular Technologies, MD). After 3 h of incubation at 37°C, we collected the supernatants and measured the absorbance at 450 nm wavelength on a multi-mode microplate reader (BioTek instruments, VT) [42]. The percent of viability was calculated as (OD_test_/OD_cell control_)×100. For human PBMCs, MTT assay was done by adding 20 µl of MTT reagent (Sigma) from 5 mg/ml stock prepared in PBS. The cells were incubated with MTT reagent for 3 h, after which 100 µl of isopropanol was added to dissolve formazan crystals. Absorbance was monitored at 570 nm wavelength on a microplate reader (BioTek instruments).

### Statistics

The results were represented as mean ± SEM for each bar graph plotted using Sigma plot v8.0 with associated p values for each treatment group compared to its controls. Statistical analysis was done using Origin 6.1 software. The significance between two groups was calculated using a two-tailed student's t-test followed by one-way analysis of variance. P<0.05 was considered to be significant.
